# P59 - Kartagener syndrome in infant – case report

**DOI:** 10.1186/2045-7022-4-S1-P114

**Published:** 2014-02-28

**Authors:** Tanya Hristova, Sirma Mileva, Milena Yankova, Ivanka Galeva, Todor Todorov

**Affiliations:** 1 Pediatric Department, Alexandrovska Hospital, Medical University Sofia, Sofia, Bulgaria

## 

Kartagener syndrome (situs inversus, sinusitis and bronchiectasis) is a rare, ciliopathic, autosomal recessive disorder that causes a defect in the action of the cilia lining the respiratory tract. Situs inversus can be seen in about 50% of cases.

We present a case of a 10-months-old girl with total situs inversus, diagnosed at birth and recurrent respiratory tract infections. The child was with repeated admissions since 5 months age. The suspicion for Kartagener's syndrome was made based on clinical presentation and radioimaging. Nasal brushing as least invasive enables observation of ciliary structures in electron microscopy. The results revealed an anomaly in the organization of the ciliary microtubules. An early diagnosis and treatment may prevent the development of bronchiectasis, which define the prognosis.

